# Fabrication of nanotweezers and their remote actuation by magnetic fields

**DOI:** 10.1038/s41598-017-00537-6

**Published:** 2017-03-27

**Authors:** Cécile Iss, Guillermo Ortiz, Alain Truong, Yanxia Hou, Thierry Livache, Roberto Calemczuk, Philippe Sabon, Eric Gautier, Stéphane Auffret, Liliana D. Buda-Prejbeanu, Nikita Strelkov, Hélène Joisten, Bernard Dieny

**Affiliations:** 1 Univ. Grenoble Alpes, INAC-SX, F-38000 Grenoble, France; 2 CEA, INAC-SX, F-38000 Grenoble, France; 3 CNRS, SX, F-38000 Grenoble, France; 4 CNRS, SYMMES, F-38000 Grenoble, France; 5 CEA, LETI, Minatec Campus, F-38000 Grenoble, France

## Abstract

A new kind of nanodevice that acts like tweezers through remote actuation by an external magnetic field is designed. Such device is meant to mechanically grab micrometric objects. The nanotweezers are built by using a top-down approach and are made of two parallelepipedic microelements, at least one of them being magnetic, bound by a flexible nanohinge. The presence of an external magnetic field induces a torque on the magnetic elements that competes with the elastic torque provided by the nanohinge. A model is established in order to evaluate the values of the balanced torques as a function of the tweezers opening angles. The results of the calculations are confronted to the expected values and validate the overall working principle of the magnetic nanotweezers.

## Introduction

Several applications in life science and biotechnology require tools for manipulating and exerting forces on micro or nanometric objects. In that regard, biocompatible magnetic nanoparticles have widely been developed and used^1–3^, owing to their ability to be actuated by external magnetic fields. The advantage of such method is that magnetic fields can penetrate human tissues in a noninvasive way. Bottom-up approaches are an efficient way to chemically produce functionalized superparamagnetic iron oxide nanoparticles in ligand matrices, and their superparamagnetic nature allows them to not agglomerate in the absence of magnetic field^4^. Functionalized magnetic nanoparticles can target specific biological entities for drug delivery, hyperthermia treatment or mechanical actuation^1^. Other methods for manipulating micro or nanoscale objects have also been explored. Notable examples are the concepts of magnetic tweezers^5–13^ and optical tweezers^14^. The former concept consists in binding a molecule to a substrate at one end and to a magnetic particle at the other end. By applying a magnetic field gradient, forces can be exerted on biomolecules such as DNA or cells to test their mechanical properties. Optical tweezers trap accelerated particles in an optical potential well. While the optical tweezers are a kind of “contactless” tweezers, past works have also established designs for “contact” tweezers^15, 16^ that are controlled by magnetic fields for grasping objects physically as well as for moving in liquid environments. They succeeded in demonstrating the possibility of transporting cargoes in the submillimeter scale through pick-and-place experiments. Devices based on the use of magnetic particles or elements along with elastic materials are also being actively developed to make programmable actuators^17^. In this article, we present a new design of magnetic tweezers prepared by a top-down approach. An advantage of such fabrication process is the fact that a vast number of nanotweezers can be produced at the same time. Moreover, with sizes between the nanometric and micrometric scales the tweezers will ultimately be able to interact with objects of comparable size such as cells, bacteria, or long chains of macromolecules. Being made of two actuable jaws, the concept of magnetic tweezer we propose is quite similar to that of real-life tweezers except that their ultimate role is to interact with micrometric species in fluids. They can potentially be an efficient way to trap or deliver molecules to targeted biological zones. Like for the nanoparticles developed in the past, self-agglomeration needs to be avoided by a proper choice of materials for the tweezers, e.g. synthetic antiferromagnetic^18, 19^ or vortex^20, 21^ particles. Due to their in-plane magnetic shape anisotropy, they can be remotely controlled by magnetic fields rather than magnetic field gradients, as seen in our previous works^22, 23^.

## Results

### Concept of the remotely actuated tweezers

**Figure 1:**
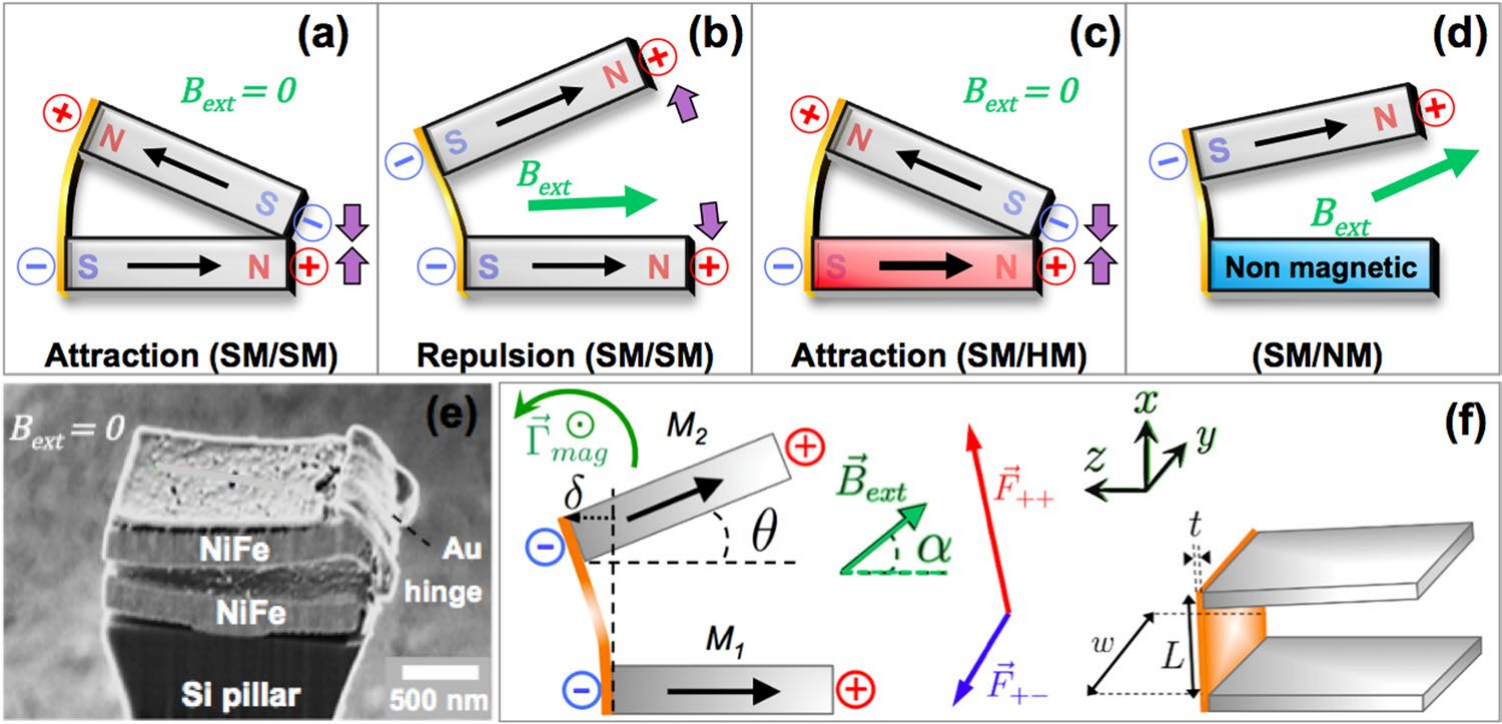
(**a**,**b**) The jaws in SM/SM tweezers attract and repulse each other in the absence and presence of an external field, respectively. (**c**) The hard magnetic jaw in the SM/HM tweezers have a stronger magnetostatic influence on the soft magnetic jaw. (**d**) The nonmagnetic jaw exerts no magnetostatic force on soft magnetic jaw. (**e**) SEM image of a SM/SM tweezer. (**f**) Coordinate system and definition of the variables that are used for the calculations.

The general concept of the remotely actuated nanotweezers is shown in Fig. 1. The nanotweezers are made of two parallelepipedic magnetic microelements, hereafter called “jaws”, that are bound by an elastic gold (Au) nanohinge. In this work, three different types of tweezers are explored. While the fabrication process is similar for each type, the tweezers can be either made of two soft magnetic jaws (SM/SM), or a soft magnetic jaw and a hard magnetic jaw (SM/HM), or a soft magnetic jaw and a nonmagnetic jaw (SM/NM), as shown in Fig. 1a–d. Each type of tweezers presents a different kind of interaction between the jaws, thus making the tweezers opening/closing process distinct in each case. For actuation purposes, it is crucial that at least one of the jaws is made of a magnet. For all three kinds of tweezers, when an external magnetic field is applied, the value of the opening angle is determined by the balance between the elastic torque stemming from the hinge and the magnetic torque coming from the external field and the magnetostatic interactions from each jaw. While the elastic torque is expressed in the same way for all tweezers, the magnetic torque is the physical quantity that will differ from one type of tweezer to another. Indeed, in the case of SM/NM tweezers, no magnetic field lines are emitted from the nonmagnetic layer, hence the soft magnetic layer will only be influenced by the external field. On the contrary, the contribution of the external field to the magnetic torque has to be added to that of the mutual magnetostatic interactions between jaws in the case of SM/SM and SM/HM tweezers. The scanning electron microscope (SEM) image in Fig. 1e shows a real SM/SM tweezer on a Si pillar, and one can clearly see the jaws bound together by a nanohinge. The coordinate system and the physical quantities related to the tweezers and the external field are defined in Fig. 1f.

**Figure 2:**
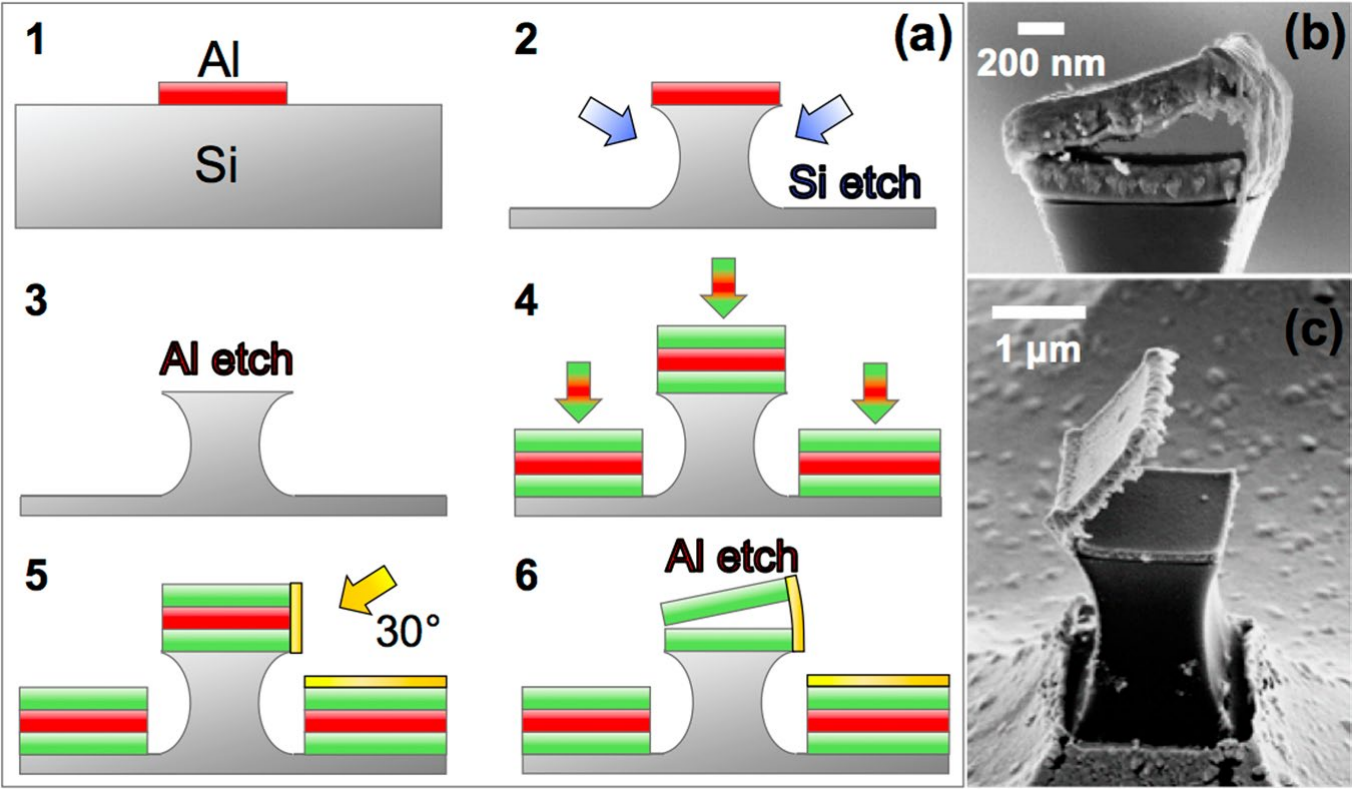
(**a**) Flowchart of the fabrication process of the tweezers. (1) The Al metal mask is patterned and deposited on the Si substrate. (2) Pillars are obtained after performing isotropic RIE. (3) The Al mask is etched away from the pillars, so that (4) the stack of metal layers that will form the base of the jaws can be deposited. (5) Then, the Au nanohinge is deposited with a 30° tilted angle, before (6) finally removing the sacrificial layer in the middle of the stack. (**b**) SEM image of a SM/SM tweezers. Both jaws are made of permalloy. (**c**) SEM image of opened SM/NM tweezers. The soft and nonmagnetic jaws are made of permalloy and chromium, respectively.

In this work, the fabrication process relies on a top-down approach (Fig. 2). The tweezers are fabricated on silicon (Si) substrates and all metal layers are deposited by electron beam evaporation. Our method here is to transfer the square-shaped mask pattern of the longitudinal section of the jaws to an aluminum (Al) hard metal mask and then, dry-etch the unprotected Si areas so that the remaining Si forms pillars that will sustain the structure of the tweezers. The Al metal mask is patterned by UV lithography on the surface of the substrate using the AZ 5214E image reversal negative tone photoresist. Once the metal mask is patterned and deposited (Fig. 2a), the unprotected parts of the wafer undergo an isotropic reactive ion etching (RIE), using sulfur hexafluoride (SF_6_) and argon (Ar) as etchant gases. Then, the main body of the tweezers is constructed by stacking three metallic layers, the topmost and lowest layers constituting the upper and lower jaw, respectively. The middle layer is an Al sacrificial layer that serves to maintain the jaws as long as they are not bound to each other by a hinge. The nanohinge is built by depositing a 20-nm-thick Au layer with a 30° oblique angle. The electron beam evaporation technique offers a relatively high directivity, which limits the amount of Au deposited on the other sides of the tweezers. In the final step, the Al sacrificial layer is chemically etched and the tweezers become free to be actuated. SEM images of the structures typically obtained after the last step are shown in Fig. 2b. The dimensions of the tweezers are 1 *μ*m × 1 *μ*m × 100 nm or 2 *μ*m × 2 *μ*m × 250 nm. Permalloy (Py) is the chosen material for the soft layers, due to its low coercivity. Nonmagnetic layers are made of chromium (Cr). Hard magnetic layers can be realized with either permanent magnets such as samarium-cobalt or neodynum-iron-boron (NdFeB) alloys, or structures with a pinned magnetization such as exchanged-biased multilayers^24–26^.

### Calculation of the equilibrium torques for each type of tweezers

For the theoretical calculations and the following experimental results, all the tweezers have their lower jaws anchored to a Si pillar and the upper jaw is always a soft magnet. Therefore, only the upper jaws can be free to move under a magnetic field. Using the notations from Fig. 1f, the angles *θ* and *α* represent the tweezers opening angle and the external magnetic field angle with respect to the immobile lower jaw plane, respectively. It is assumed that the deformation of the Au nanohinge is invariant in the *y* direction and occurs only in the (*xOz*) plane. Therefore, the hinge can be divided into several slender portions of length *L*, thickness *t* and width d*y*. One can thus apply the Euler-Bernoulli theory^27^ for end-loaded beams on each portion d*y* and integrate over the entire width *w*, which results in the following relation between the hinge bending moment and the deflection *δ*: Γ_hinge_ = *Ewt* ^3^ *δ*/6*L* ^2^, where *E* is the Young’s modulus of the hinge, *w* is the total width of the hinge and *L* the length of the mobile part of the hinge. With the approximation *δ*/*L* ≃ sin*θ*, one can express Γ_hinge_ as a function of *θ*:

1 $${{\rm{\Gamma }}}_{{\rm{hinge}}}=\frac{Ew{t}^{3}}{6L}\,\sin \,\theta =K\,\sin \,\theta \mathrm{.}$$

**Figure 3:**
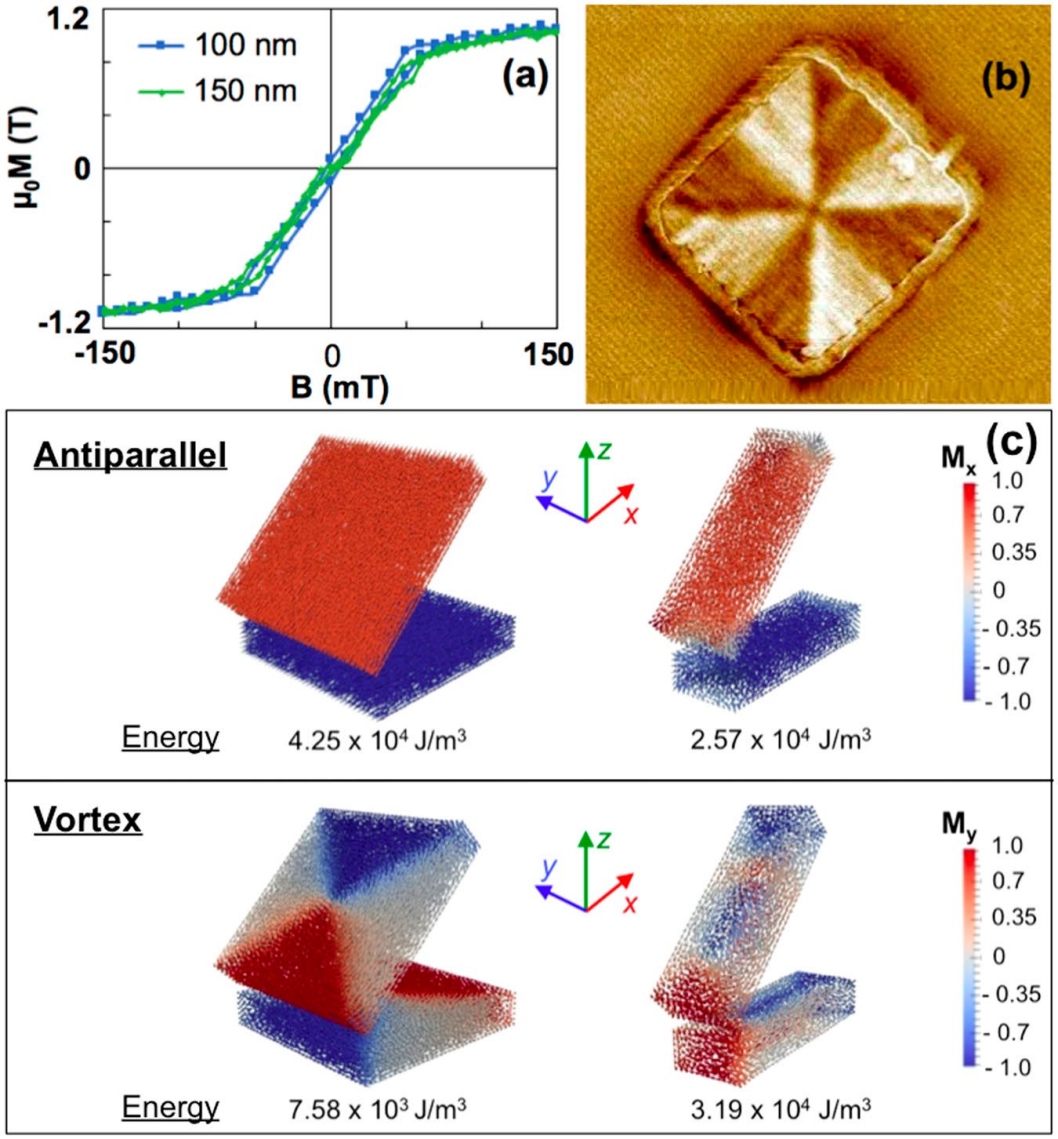
(**a**) Magnetic hysteresis curves of permalloy square-shaped microelements. The magnetization is a quasilinear function of the external field in the unsaturated region. (**b**) MFM image of a soft magnetic jaw (2 *μ*m × 2 *μ*m × 250 nm) at remanence showing a vortex structure. (**c**) Micromagnetic simulations at zero field and with a tweezers opening of 45° for different jaw geometries (square and elongated) showing different metastable magnetic configurations. The magnetization is normalized. The energy of each state shows that while the antiparallel configuration can exist for two square-shaped jaws, the two-vortex-state is energetically more favorable. In contrast, elongated shapes tend to stabilize the antiparallel configuration.

*K* has the dimension of a torque and can be seen as the nanohinge stiffness coefficient. Regarding the magnetic properties of the Py soft jaws, it is clear from Fig. 3a that the in-plane magnetic hysteresis curves show very small remanence and coercivity. A magnetic force microscopy scan performed on one Py soft jaw (Fig. 3b) at remanence indicates that the soft jaw magnetization has a vortex structure. Consequently, the behavior of soft jaws under a magnetic field follows the assumptions that the magnetization responds in a quasilinear and reversible way to the magnetic field below saturation, and that the in-plane magnetic shape anisotropy is sufficiently large so that the net magnetization vector remains in the plane of the jaw.

The vortex state is the most stable magnetic configuration for an isolated square-shaped soft magnetic microelement. However, when two of those elements are put in proximity to each other as in SM/SM tweezers, dipolar interactions can actually favor a magnetization alignment despite the absence of field. The geometry of the jaw has also an influence on the nature of the most stable configuration adopted by the two interacting jaws. In order to illustrate this statement, micromagnetic simulations were performed using Magpar^28^, a finite element micromagnetics package. Figure 3c represents the case of square-shaped elements and that of elongated elements, for which the width is one third of its length. The materials parameters correspond to that of permalloy, i.e. *M*_S_ = 1 T, *A*_ex_ = 1.05 × 10^−11^ J.m^−1^, *K*_u_ = 0 J.m^−3^, which correspond to the saturation magnetization, the exchange constant, and the magnetocrystalline anisotropy constant, respectively. After initializing the simulation in a vortex configuration, we found that in the case of square-shaped elements, the system reaches an antiparallel state and then relaxes to a two-vortex state, as shown by the values of magnetic state energy in Fig. 3c. On the contrary, the elongated geometry favors the antiparallel magnetization configuration. The conclusion of this micromagnetic simulation is that the combination of dipolar coupling and shape anisotropy tends to increase the stability of the antiparallel state. Such case of stable antiparallel magnetization alignement is depicted in Fig. 1a.

**Figure 4:**
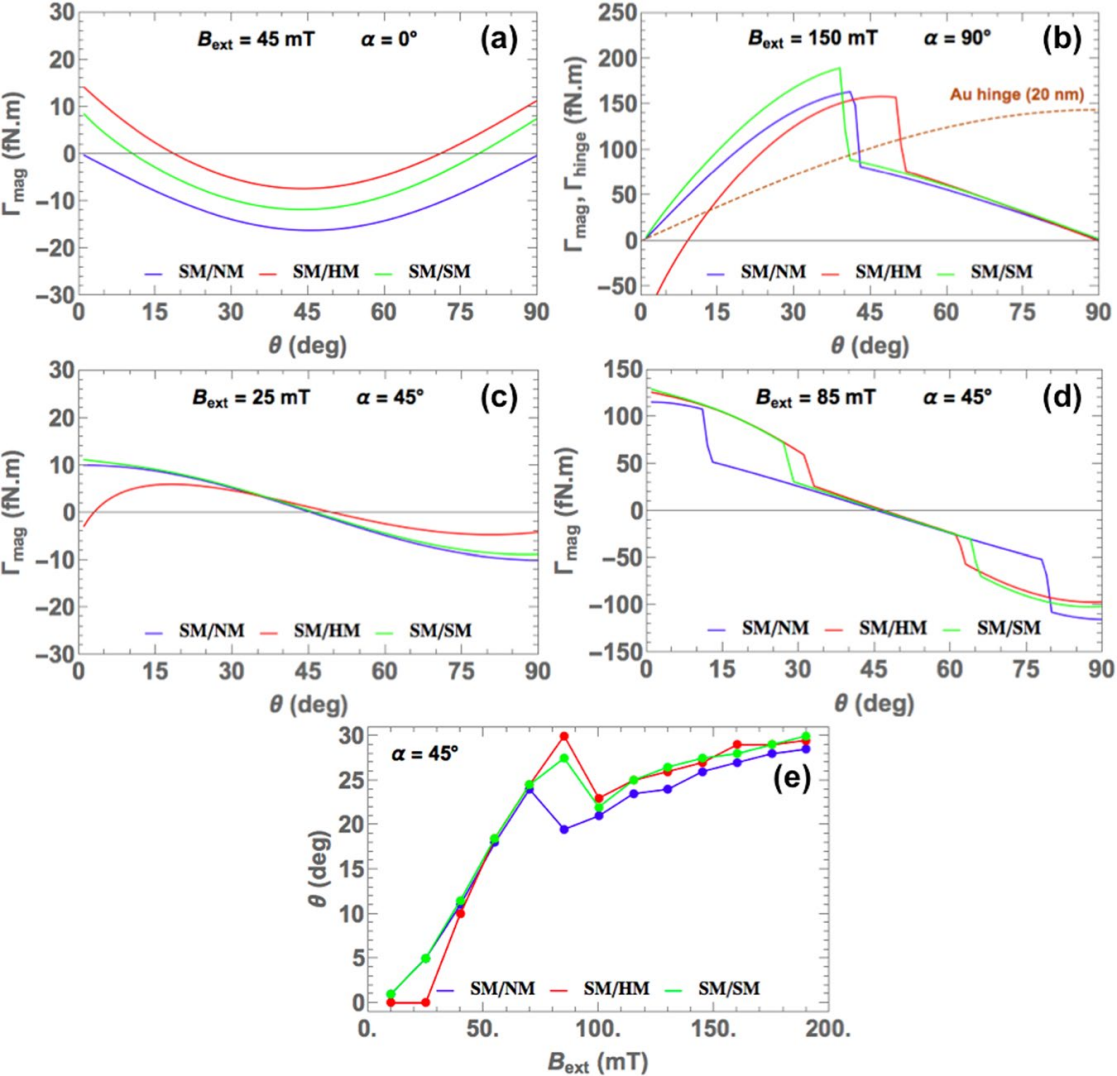
For the three types of tweezers, the magnetic torque is calculated as a function of opening angle for different field angles (**a**) *α* = 0°, (**b**) *α* = 90°, (**c**,**d**) *α* = 45°. The elastic torque stemming from the Au nanohinge is shown only in (**b**). (**e**) The equilibrium opening angle at *α* = 45° is determined by intersecting the magnetic torque and elastic torque curves for several amplitudes of magnetic field.

In this paragraph, we estimate the magnetic and mechanical torques on square-shaped tweezers. The magnetic torque exerted on the upper jaw is expressed as a function of the total magnetic energy *E*_t_: Γ_mag_ = ∂*E*_t_/∂*θ*, where *E*_t_ = −**M** *V* · (**B**_ext_ + **B**_m_), with **M**, *V*, **B**_ext_ and **B**_m_ being the magnetization, the volume of the jaw, the external field and the field generated by the lower jaw, respectively. **B**_m_ is directly related to the surface magnetic charge distribution on the jaws. Since the field is not necessarily applied along one of the jaw’s plane, only the component of the field in the plane of each jaw counts. The absence of jaw-to-jaw interactions in SM/NM tweezers highlights the role played by the magnetostatic interactions in the tweezers made of two magnetic jaws. In SM/HM tweezers, the stray field produced by the hard layer will hardly vary with the strength and direction of the applied field, so magnetostatic interactions will also exist at zero field. In contrast, in SM/SM structures, the magnetic domain configurations of both soft layers are subjected to change under the applied field. As a result, at zero field, since the soft magnetic square elements have a vortex configuration, the magnetic flux closure from the lower jaw prevents its stray field from radiating to the upper jaw although it has been shown that in the case of square-shaped microelements with vortices, a stray field still arises from the Néel domain walls at the square diagonals^29, 30^. When the magnitude of the in-plane component of the field increases in each soft layer, vortex annihilation eventually occurs and the layers saturate, thus the material exits the linear regime. The variations of Γ_mag_, exerted on the upper jaw, as a function of *θ* are represented in Fig. 4a–d for different magnitudes of *B*_ext_, and field angle *α*. In the case of SM/NM tweezers, the upper jaw is only influenced by the external field and tends to align with its direction. When *α* = 0°, deviating the upper jaw from its *θ* = 0° position will favor negative magnetic torque, i.e. tweezer closing, with a minimum at *θ* = 45°.

Similar behaviors are predicted for SM/HM and SM/SM tweezers but the magnetostatic influence from the lower jaw favors the tweezers opening at angles close to 0° or 90°. Jumps in the magnetic torques occur at values of opening angles, for which entry in the saturated state or reentry in the unsaturated state happen (Fig. 4b,d). One must note that the jumps occur at different opening angles in the three types of tweezers. The presence of magnetostatic interactions in addition to the external field in the SM/SM and SM/HM tweezers affects the opening/closing process of the tweezers. Consequently, the magnetization of the upper jaw does not saturate at the same value of tweezer opening angle in each case. Figure 4b also includes the variations of Γ_hinge_, calculated for a 20-nm-thick Au hinge with *E* = 20 GPa. Au layers deposited by e-beam evaporation can have lower Young’s moduli than the bulk material^31^. Torque balancing is found by intersecting the curves of Γ_mag_ and Γ_hinge_; Fig. 4e plots the values of equilibrium opening angle with respect to a **B**_ext_ applied at 45°. The significant difference between the results obtained for SM/NM structures and two-magnetic-jaw structures again illustrates the effects of the presence of magnetostatic interactions. It is interesting to point out that when the field is applied perpendicularly to the lower jaw plane, two positions of torque equilibrium exist for SM/HM tweezers (Fig. 4b).

The value of Young’s modulus in the abovementioned calculation is chosen to be smaller than the value corresponding to e-beam-deposited Au layers (55–62 GPa)^31^, which is itself smaller than the bulk value (79 GPa). The oblique incidence angle of the Au deposition leads to self-shadowing effects at the nanometric scale, due to the columnar growth mode of the Au islands. Therefore, the density of the hinge can be drastically reduced compared to a layer that would have been grown with a normal incidence. In order to account for this difference in density due to the oblique incidence, the numerical value of the Young’s modulus of the hinge is adapted in this study, although it proves difficult to determine its precise value. An order of magnitude of about half the value mentioned in the past work^31^ yields mechanical torques that are compatible with the magnetic torques estimated above and the mechanical torques we obtain with the experiments described in the following section.

Although it is expected from the considered application of the tweezers that the magnetic and elastic torques are the main operators of the tweezers actuation, effects of intermolecular forces and quantum effects can arise given the nanometric to micrometric size of the tweezers. Those effects can indeed contribute to keeping the jaws closed by mutual attraction. We estimated that the effects of the Casimir effect and Van der Waals forces have a magnitude of the order of 1 nN, which is two orders of magnitude lower than the forces due to the magnetic field. Therefore, the presence of a magnetic field can always overcome the Casimir effect and the Van der Waals forces. However, in the absence of magnetic field, mutual attraction between the jaws can occur when the upper jaw is close enough to the lower jaw. In this kind of situation, the jaws stick together but a mechanical actuation from an AFM tip is sufficient to reopen the tweezer, thus enabling further actuations with an external magnetic field.

## Discussion

**Figure 5:**
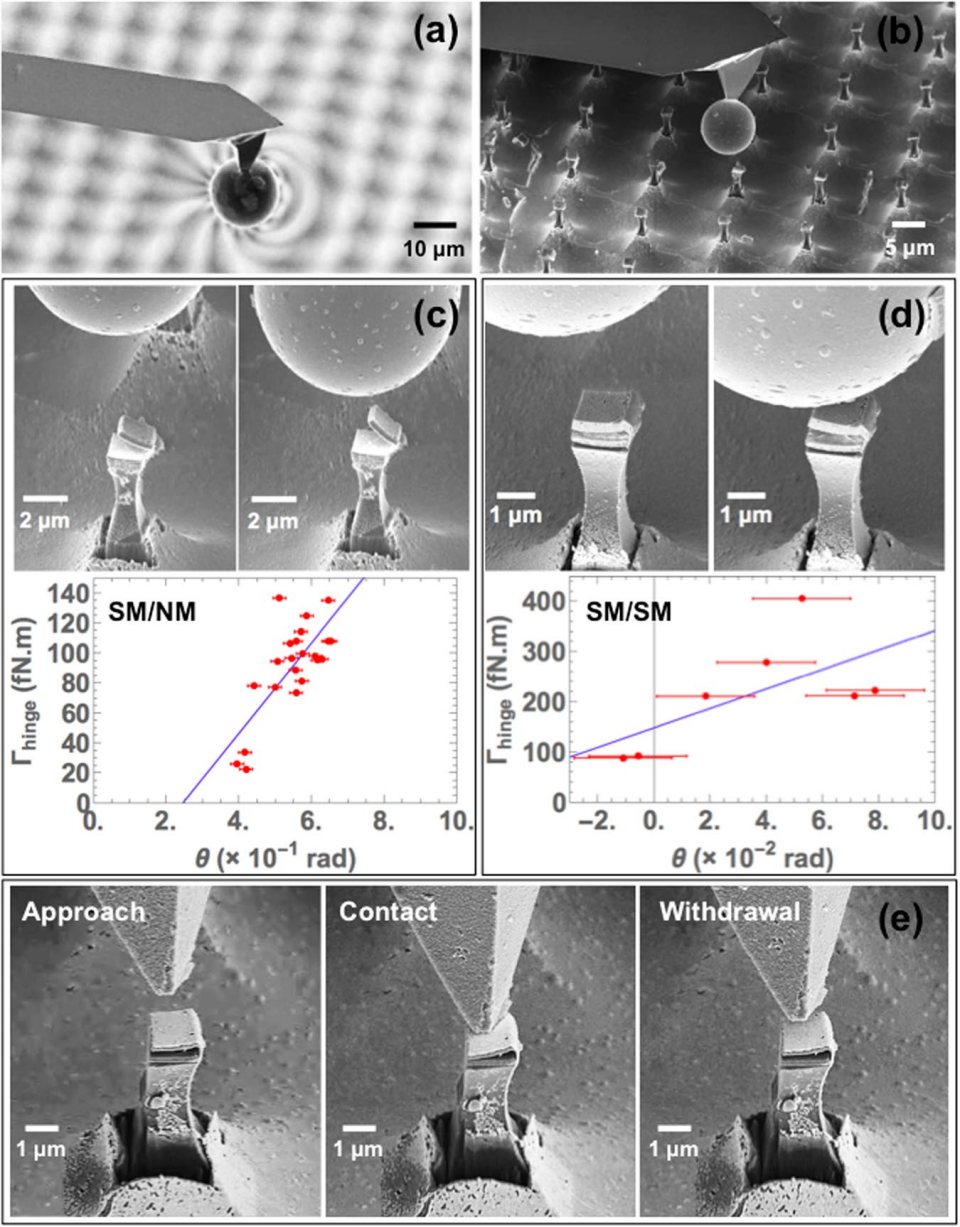
(**a**) FIB image of the magnetic microsphere mounted on the AFM tip. The field lines cause a deflection of the Ga^+^ ions, which is very localized around the sphere and does not disturb the image acquisition as much as in the case of electrons. (**b**) Image of the probe targeting one the tweezers in the array of tweezers. Values of Γ_hinge_ are calculated and plotted as a function of the measured tweezer opening angle in the case of a (**c**) SM/NM structure and a (**d**) SM/SM structure. (**e**) Test of the mechanical properties of the Au nanohinge with an AFM tip on SM/SM tweezers.

In order to test the ability of the tweezers to be actuated by a magnetic field, direct observation is performed inside a SEM chamber. The magnetic field is provided by a homemade probe (see details in the Methods section), which is made by soldering a NdFeB hard magnetic microsphere to an atomic force microscope (AFM) tip (Fig. 5a,b). Real-time actuation can be observed when the field lines encounter the upper jaw of a tweezer. Introducing a magnetic field in the SEM chamber can distort the path of the electrons, thus potentially rendering bad image acquisition. However, the SEM system also incorporates a focused ion beam (FIB) setup, which improves the quality of the images, since ions are not as easily deflected by a magnetic field as electrons. Moreover, the magnetic microsphere has a sufficiently small size so that its generated magnetic field is localized in the vicinity of a single tweezer, thus introducing minimal disturbance. It is important to emphasize that the images in Fig. 2b,c were acquired by using electron beams only. The opened tweezers shown in Fig. 2c is kept opened, not by an applied magnetic field, but because of the presence of a residual mechanical stress in the Au hinge. Depending on the thermal history of each hinge, the stress can be either tensile or compressive. The effects of the residual stress are visible in the results of the experimental data, when measuring tweezers opening angles.

The results of the characterization of the tweezers are presented in Fig. 5 for the cases of SM/NM and SM/SM structures. The characterization is done as follows: depending on the position of the microsphere relatively to the tweezer, the opening angle will have a certain value. This angle is determined from the SEM measurements, with an estimated uncertainty of ±1°. For each value of opening angle obtained by SEM, the magnetic torque exerted on the upper jaw by the magnetic microsphere and the lower jaw is calculated. Considering that the magnetic torque Γ_mag_ is in equilibrium with the elastic torque Γ_hinge_, a linear relationship is expected between the calculated torque and the measured equilibrium opening angle of the tweezers. Indeed, the measured values of *θ* are included in a range that allows to regard Γ_hinge_ as a linear function of *θ*. Therefore, calculating the slope of that plot ultimately leads to the determination of the thickness of the nanohinge. The field generated by the hard magnetic microsphere is modeled as a dipole: ${\bf{B}}=\frac{{\mu }_{0}}{4\pi {r}^{3}}[\mathrm{3(}{\bf{m}}\cdot {\bf{r}})\cdot {\bf{r}}-{\bf{m}}]$, where **r** is the distance from the center of the sphere and **m** is the dipolar moment. For a given pair of jaws, this expression is added to the contribution of the magnetostatic interaction from the lower jaw and then integrated over each point in the upper jaw. The numerical values of the equilibrium torque are deduced, as can be seen in Fig. 5c,d. The diameter of the sphere is 10 *μ*m and its remanent magnetization is measured by vibrating sample magnetometer (VSM) to be 5.8 × 10^5^ A/m, which is about half of the saturation value, meaning that the sphere is polycrystalline with random grain anisotropy orientation. The equilibrium torque calculations yield a significant dispersion of the points. Nevertheless an estimation of the hinge stiffness can be made by linear interpolation. The slopes corresponding to each linear fits are: *K*_SM/NM_ = 3.02 × 10^−13^ N.m for the SM/NM tweezer, and *K*_SM/NM_ = 1.95 × 10^−12^ N.m for the SM/SM tweezer. By using equation (1), the effective thicknesses of the Au nanohinge in each case are calculated to be *t*_SM/NM_ = 25.6 nm and *t*_SM/NM_ = 47.7 nm. While the value of nanohinge thickness determined for the SM/NM tweezers is relatively close to the real deposited thickness, the effective thickness found for SM/SM tweezer is approximately twice the expected value. Both types of tweezers had their nanohinge deposited with 30° oblique incidence with an expected thickness of 20 nm. Knowing that Py/Al/Py trilayer stacks tend to present rougher surfaces than Py/Al/Cr stacks, one would then expect that the thickness of the Au layer shows higher fluctuations in the case of SM/SM tweezers. This can account for the discrepancy between effective and nominal hinge thickness observed for the SM/SM tweezer. The oblique angle deposition of the Au layer, combined with the effects of the roughness of the previous metal layers, can indeed cause the Au atoms to clump up and form regions with inhomogeneous thicknesses^32^. The growth method can also favor nanometer-scale self-shadowing in the Au layer by nanoislands^33^. As Γ_hinge_ varies as the third power of the hinge thickness, porosity and thickness fluctuations will locally change the elastic torque and affect the overall stiffness of the hinge. To explain the increased stiffness of the Au nanohinge in the SM/SM structure, the following model is proposed: we consider an ideal Au layer with a nominal thickness of *t*_nom_ = 20 nm grown in a two-dimensional mode and the real Au layer that is effectively deposited with a thickness *t*_eff_ in a three-dimensional mode. In principle, both layer have the same volume, since they only differ by their growth modes. According to the expression of the stiffness coefficient defined in equation (1), the stiffness of the hinge varies as the third power of its thickness. Let us then we compare two Au layers used as hinges of same nominal volumes, one of them presenting a perfectly uniform thickness and the other having a large roughness characterized by thin (or even empty) regions coexisting with thicker regions. Since the two layers have the same volume, the thicker regions in the nonuniform layer can be substantially thicker than the originally intended thickness of 20 nm. Due to the cubic thickness dependence of the hinge stiffness as seen in equation (1), those thick regions contribute in increasing the effective stiffness of the hinge compared to the case where the hinge has a uniform thickness. We define *τ* as the proportion of void in the real nanohinge. By conservation of volume, the nominal width and thickness are related to the effective dimensions as follows, *w*_eff_ = (1 − *τ*)*w*_nom_ and *t*_nom_ = (1 − *τ*)*t*_eff_. Therefore, the ratio of stiffness coefficient is expressed as *K*_nom_/*K*_eff_ = (1 − *τ*)^2^ and leads to a void proportion of about 30% and 70% for the SM/NM and SM/SM tweezers, respectively. According to this model, the discrepancy between the values of equilibrium torque, hence Au thickness, obtained by the linear fits and the expected values actually represents a change in effective stiffness of the deposited nanohinges. Thus, according to this model, a higher void proportion leads to an enhancement of stiffness. A possible way to avoid the inhomogeneity in Au thickness is to reduce the roughness of each of the previous metal layers by Ar plasma etching. The reversibility of the nanohinge deformation was also tested by applying a mechanical pressure on the surface of the upper jaw of a tweezer with an AFM tip. The real-time elastic response of the tweezer is recorded by SEM, and evidently shows the contribution of the elastic torque from the gold nanohinge, which tends to bring the upper jaw back to its initial position (Fig. 5e).

It is also important to notice that according to equation (1), the linear fits in Fig. 5 should in principle cross the zero point. However, this is not the case. Indeed, as explained earlier, some residual stress arises within the Au hinge and depending on the thermal history of each hinge, this stress can be either tensile or compressive, as mentioned before. This explains why the curves in Fig. 5c,d do not cross the zero point. In the case of Fig. 5c, the angle value at zero torque is positive, which means that the residual stress tends to open the tweezers. In contrast, the graph in Fig. 5d represents a case where the residual stress tends to close the tweezers. While we can optimize the fabrication process to remove the porosity of the Au hinge, residual mechanical stress will always be present. But analyses such as the one presented in Fig. 5 makes it possible to characterize the effect of such stress on the tweezers.

In summary, the real-time actuation of the tweezers by moving the NdFeB microsphere and the torque calculations support the proof of concept of the nanotweezers. In this work, experimental results are shown only for SM/NM and SM/SM tweezers. Realizing hard layers in SM/HM tweezers require additional technical steps. The goal of this work was to present the working principle of the magnetic nanotweezers and showing the actuation of anchored SM/NM and SM/SM tweezers is sufficient for that purpose. SM/HM tweezers will actually be of significant importance for applications in fluids, i.e. when they are released in a solution, because the hard magnetic layer will allow to orient the tweezers depending on the external field. Both jaws will tend to orient in a way that minimizes the Zeeman energy, but the harder layer would always follow the field thus orienting the tweezer. So, as a future prospect, the tweezers could be released in a solution, be displaced in the fluid by application of a field gradient and oriented by application of a magnetic field. The effect of the magnetic field would therefore be twofold: it would control the opening/closing movements of the tweezers and orient them during their displacement. Such remote actuation of the tweezers in a fluid would make it an attractive tool for manipulating, grabbing micro or nanometric objects. With a proper surface functionalization, tweezers can be designed to target specific biological environments or catch biomolecules.

## Methods

The probe that was used in the FIB experiment is homemade and involves several preparation steps. The hard magnetic NdFeB microsphere was obtained by drying a liquid containing NdFeB powder having a relatively high dispersion in particle size, with a mean diameter of 50 *μ*m, on a silicon wafer. The more the liquid wets the Si wafer, the more dispersed the spheres will be, thus facilitating the selection of a sphere with a proper diameter, given the size of the tweezers. The dispersed spheres are observed in a SEM chamber equipped with FIB. A soft contact is made between the selected sphere and an AFM tip, then the two elements are welded to each other with a tungsten bond, which is done by applying gas-assisted process. The gas used here is tungsten hexacarbonyl (W(CO)_6_). Once the probe has successfully been constructed, it is taken out of the SEM chamber in order to be magnetized between the poles of the electromagnets of a VSM (magnetic field of 1.7 T), prior to the tweezers actuation experiments.

Removing the Al sacrificial layer between the two jaws at the end of the tweezers fabrication process is actually a challenging process because one needs to dissolve this layer without altering any of the jaws. The tweezers are dipped in an Al etchant solution for 20 min. The toughest step is the drying step, since capillary forces due to the removal of the liquid can exert forces on the jaws and therefore break the hinges. Capillary forces occur when a liquid meniscus forms between the jaws. The formation of such meniscus can however be minimized by using a supercritical dryer.

No effect of electrostatic forces have been observed during SEM characterization. The tweezers themselves do not charge during the SEM observations since they are metallic and not made on insulating materials. Charge-up phenomena during SEM observations usually occur progressively and alter the image over time. However in our case, no distortion and no bleaching of certain areas were observed. The SEM images are very stable (as long as no magnetic field is introduced). The presence of electrostatic forces would indeed contribute to actuating tweezers, however such actuation was not observed. The tweezers remain in their state even after long observations with the electron beam. The pillars holding the tweezers are made of Si, which is not perfectly conducting but it is not insulating enough for charges to accumulate.

## Electronic supplementary material

Supplementary Video Legends

Magnetic actuation of tweezers in a SEM chamber
