# Supplementary material for: Fabrication of nanotweezers and their remote actuation by magnetic fields
Source: Sci Rep. 2017 Mar 27;7:451. doi: 10.1038/s41598-017-00537-6 (PMC5428679; doi:10.1038/s41598-017-00537-6)
Supplement: Supplementary file 1 — Supplementary Video Legends [file 41598_2017_537_MOESM1_ESM.pdf]

Fabrication of nanotweezers and their remote actuation by magnetic fields

Cécile Iss[1,2,3], Guillermo Ortiz[1,2,3], Alain Truong[1,2,3], Yanxia Hou[1,2,3,4], Thierry Livache[1,2,3], Roberto Calemczuk[1,2,3], Philippe Sabon[1,2,3], Eric Gautier[1,2,3], Stéphane Auffret[1,2,3], Liliana Buda-Prejbeanu[1,2,3], Nikita Strelkov[1,2,3], Hélène Joisten[1,2,3,5], and Bernard Dieny[1,2,3,*]

[1]Univ. Grenoble Alpes, INAC-SX, F-38000,, Grenoble, France
[2]CEA, INAC-SX, F-38000, Grenoble, France
[3]CNRS, SX, F-38000, Grenoble, France
[4]CNRS, SYMMES, F-38000, Grenoble, France
[5]CEA, LETI, Minatec Campus, F-38000, Grenoble, France
*Correspondence and requests for materials should be addressed to B. D. (email: bernard.dieny@cea.fr)

Supplementary video legends:

Supplementary video 1: real-time actuation of tweezers in a SEM chamber. This video corresponds to the experiment shown in Fig. 5c. One can see that the tweezers are actuated as the position of the hard magnetic microsphere is changed.
